# The impact of polydioxanone (PDS) foil thickness on reconstruction of the orbital geometry after isolated orbital floor fractures

**DOI:** 10.1007/s00068-024-02585-w

**Published:** 2024-06-28

**Authors:** Philipp Winnand, Mark Ooms, Nassim Ayoub, Daniel Schick, Felix Paulßen von Beck, Frank Hölzle, Thomas Mücke, Ali Modabber

**Affiliations:** 1 Department of Oral and Maxillofacial Surgery, University Hospital RWTH Aachen, Pauwelsstraße 30, D-52074 Aachen, Germany; 2 Department of Oral and Maxillofacial Surgery, Helios St. Josefshospital Uerdingen, Kurfürstenstraße 69, D-47829 Krefeld, Germany; 3 Oral and Maxillofacial Surgery Kleve, Triftstraße 95-97, D-47533 Kleve, Germany

**Keywords:** Cadaver study, Orbital floor reconstruction, Orbital floor repair, Orbital fracture, Orbital geometry, Orbital height, Orbital volume, Polydioxanone (PDS) foil, Resorbable orbital implant

## Abstract

**Purpose:**

The orbital floor is frequently involved in head trauma. Current evidence on the use of reconstruction materials for orbital floor repair is inconclusive. Accordingly, this study aimed to compare the impact of polydioxanone (PDS) foil thickness on reconstruction of the orbital geometry after isolated orbital floor fractures.

**Methods:**

Standardized isolated orbital floor fractures were symmetrically created in 11 cadaver heads that provided 22 orbits. PDS foils with thicknesses of 0.25–0.5 mm were inserted. Computed tomography (CT) scans of the native, fractured, and reconstructed orbits were obtained, and orbital volume, orbital height, and foil bending were measured.

**Results:**

Orbital volume and height significantly (*p* < 0.01) increased after the creation of isolated orbital floor fractures and significantly (*p* = 0.001) decreased with overcorrection of the orbital geometry after orbital floor reconstruction with PDS 0.25 mm or PDS 0.5 mm. The orbital geometry reconstruction rate did not differ significantly with respect to foil thickness. However, compared to PDS 0.5 mm, the use of PDS 0.25 mm resulted in quantitatively higher reconstructive accuracy and a restored orbital volume that did not significantly differ from the initial volume.

**Conclusion:**

Orbital floors subjected to isolated fractures were successfully reconstructed using PDS regardless of foil thickness, with overcorrection of the orbital geometry. Due to its lower flexural stiffness, PDS 0.25 mm appeared to provide more accurate orbital geometry reconstruction than PDS 0.5 mm, although no significant difference in reconstructive accuracy between PDS 0.25 mm and PDS 0.5 mm was observed in this cadaveric study.

## Introduction

The orbital walls are commonly involved in maxillo-facial trauma, either as isolated blowout fractures or in combination with other fractures of the facial skeleton [1]. Hydraulic forces, which increase the pressure within the orbit, and buckling mechanisms, which transmit direct force to the orbit, contribute to fractures of the orbital walls that are sites of least resistance [2]. Diplopia, enophthalmos, and muscle entrapment with impaired bulbus motility are clinical indications for surgical orbital floor reconstruction [3]. Surgical reduction of the herniated orbital tissue and subsequent coverage of the orbital floor defect are performed to restore the orbital geometry, specifically the orbital volume and height [4, 5].

Reconstruction of the orbital floor can be performed with autologous, resorbable alloplastic, and nonresorbable alloplastic materials. Ideal reconstruction materials exhibit the following characteristics: resistance to infection, low reactivity, self-dissolution, osteoconduction, cost-effectiveness, availability, inhibition of capsule formation, and a half-life that allows bone regeneration [6–8]. Since the ideal material for orbital floor reconstruction has not yet been identified and no consensus has been reached on standardized treatment algorithms, surgeons choose the reconstruction material on a case-by-case basis.

The German AWMF guideline recommends the use of resorbable foils for small-sized defects [9]. Polydioxanone (PDS) foils have been in use for more than 30 years [10]. PDS loses half of its strength after three weeks and is completely resorbed after six months [11]. PDS foils are available with 0.15 mm, 0.25 mm, and 0.5 mm thicknesses, and are designed to provide stability to the orbital floor via an autologous soft tissue scar plate that forms as the PDS foil material degrades [10, 11]. Orbital floor reconstruction with PDS foils is associated with low rates of enophthalmos, infraorbital hypesthesia, and bulbar motility disorders and guarantees good long-term results [4, 5, 12–15]. However, the indication for orbital floor reconstruction with PDS foils is inconsistent in the literature. While most papers limit the indication for PDS foil insertion to a maximum fracture area of 2.5–3.0 cm^2^ [16–18], PDS foils have also been used for a median fracture size of 4.32 cm^2^ [5] and extended fracture areas of up to 10.19 cm^2^ [19].

The biomechanical principles underlying the effects of PDS foils and their characteristics over time have been studied by others [20–22], and their findings suggest that the thickness of the PDS foils used exceeds the actual need for adequate support of the orbital contents [21, 23, 24]. Although the use of PDS foils with different thicknesses for orbital floor reconstruction has been studied, no study has sufficiently investigated the use of PDS foils with different thicknesses for standardized fracture sizes. Hence, this human cadaver-based study aimed to compare the impact of PDS foil thickness on the reconstruction of the orbital geometry after isolated orbital floor fractures.

## Materials and methods

### Materials

**Fig. 1:**
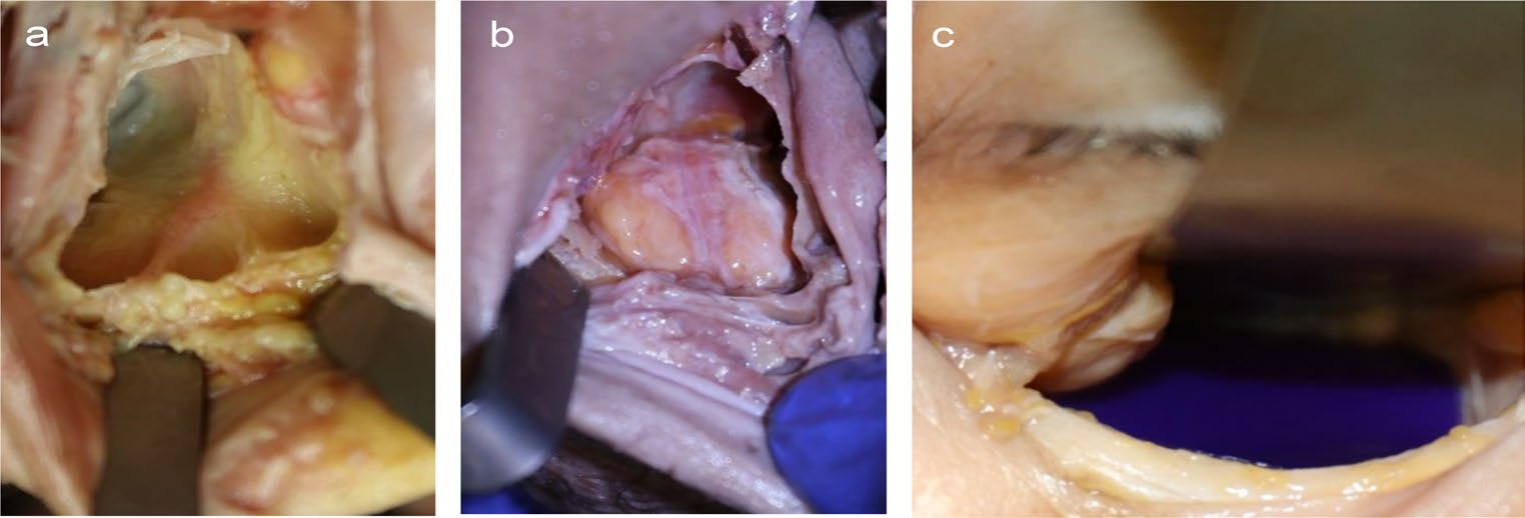
Representative examples of the fractured and repositioned orbital floors examined in this study. An intact right-sided (**a**) and a fractured left-sided (**b**) orbital floor, shown from below. Insertion of PDS foil into a left-sided orbit (**c**)

A total of 11 fresh frozen cadaver heads, each providing one left and one right intact orbit, were included in the study. The orbital floor was exposed via an intraoral approach (Fig. 1a). Standardized isolated orbital floor defects were symmetrically created by piezosurgical removal of the orbital floor (Fig. 1b). The posterior ledge, corresponding to the dorsal tip of the orbit, and the inferior-medial strut, as a bony connection between the medial and caudal orbital walls, were preserved. The bulbus was inflated with 0.9% NaCl to 30 g, corresponding to the weight of the orbital contents [21, 23, 24], to simulate orbital tissue herniation into the maxillary sinus. The fractures were exposed via an infraorbital approach, and repositioning of the herniated orbital tissue was performed with a periosteal elevator. PDS foils (Ethicon, Norderstedt, Germany) with 0.25 mm (ZX3) or 0.50 mm (ZX4) thicknesses were placed on the intact bony buttresses into the fractured orbits (Fig. 1c).

Computed tomography (CT) scans of the native, fractured, and reconstructed orbits were acquired using a Siemens Somatom Definition Flash scanner at a slice thickness of 1 mm.

### Methods

#### Image processing

The digitalized cadaver head data were exported from the Picture Archiving and Communication System (PACS) and converted to the Digital Imaging and Communications in Medicine (DICOM) format. The head images were aligned to the median sagittal plane and Frankfurt horizontal.

#### Orbital measurements

The digitalized cadaver head images were imported into the Brainlab iPlan 3.0 ENT program (Brainlab AG, Munich, Germany) for measurement of the orbital volume and orbital defect area using two-dimensional and three-dimensional measurement tools.

**Fig. 2:**
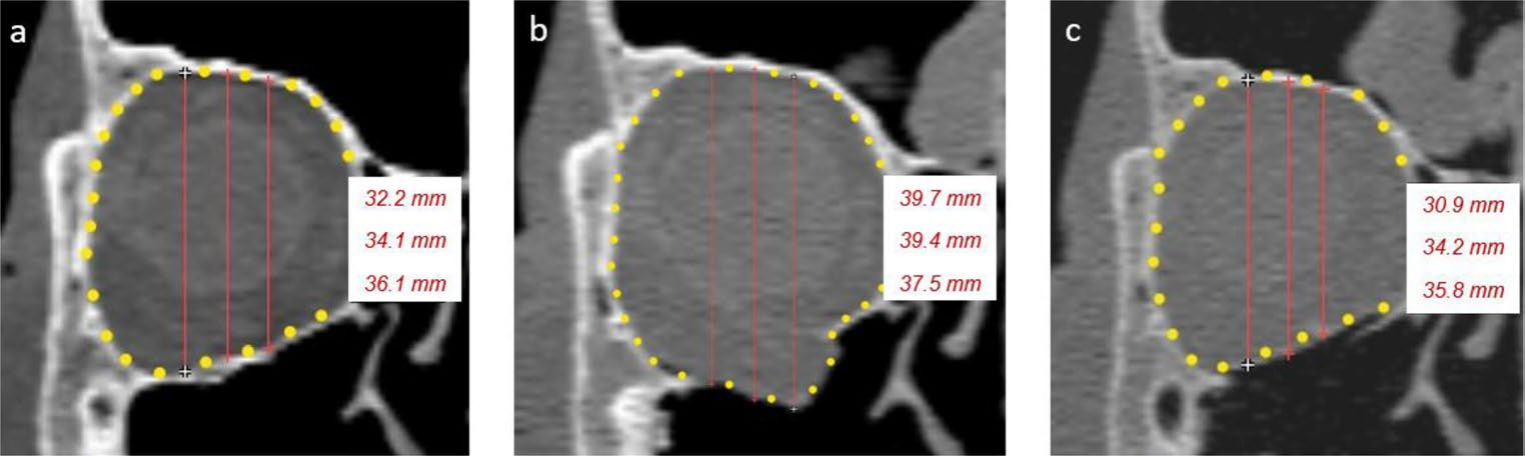
Measurement of orbital geometry. Measurement of orbital height (red lines) and orbital volume (yellow dots) in native (**a**), fractured (**b**), and reconstructed (**c**) right-sided orbits

Orbital height and foil bending were measured using ProPlan CMF 3.0.1 software (DePuy Synthes, Solothrun, Switzerland, and Materialise, Leuven, Belgium). Orbital height was measured using a special algorithm that could be performed on bony reference points without fracture involvement. In the sagittal plane, approaching from the lateral side, the layer in which the superior orbital fissure was first visible was selected. In this sagittal plane, the orbital floor length—from the infraorbital rim to the posterior ledge—was measured and bisected. At the bisection point of the orbital floor, the image plane was changed to a coronal view. In the coronal image layer, the orbital height was measured in the center and 5 mm medially and laterally. Matching sequences were identified so that orbital height was measured in the same image sections for the native, fractured, and reconstructed orbits (Fig. 2).

**Fig. 3:**
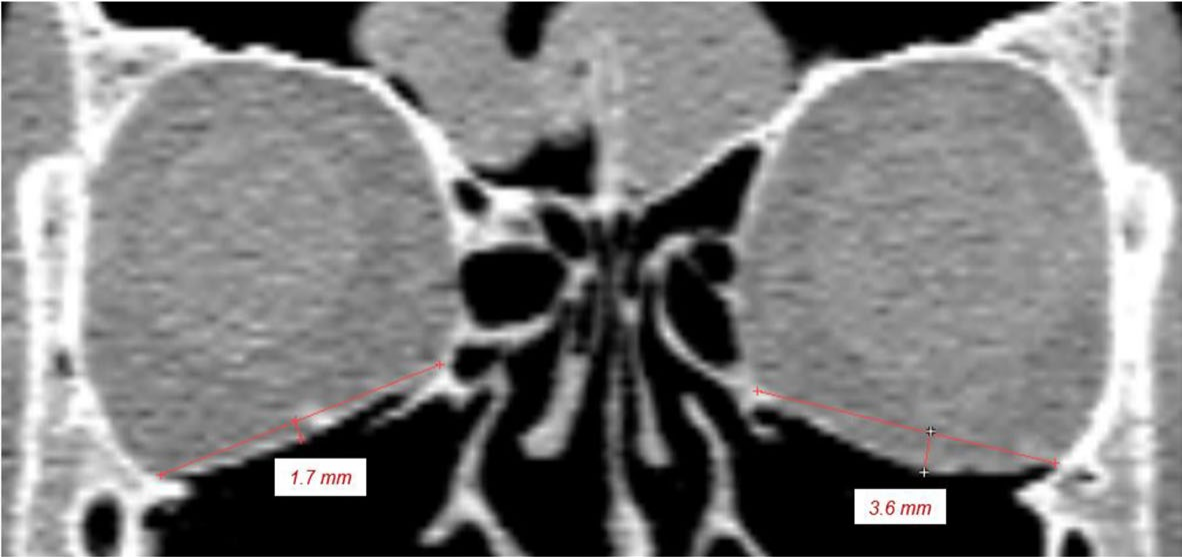
Foil bending. Bending of PDS 0.25 mm (left) and PDS 0.5 mm (right)

Foil bending was calculated perpendicular to the bony supports of the foils in the coronal image section with the greatest bending of the PDS foil (Fig. 3).

#### Statistical analysis

The orbital characteristics (i.e., orbital defect area, orbital floor area, orbital volume, and orbital height) are shown as median values with interquartile ranges. The differences between the baseline characteristics of the orbits reconstructed with PDS 0.25 mm or PDS 0.5 mm were analyzed using the Mann–Whitney test. The differences between the initial, fractured, and reconstructed orbital volumes and heights were analyzed using the Wilcoxon signed-rank test.

As per Wi et al. [25], the reconstruction rates of the orbital volume and orbital height were calculated using the formula (1 − (A − B)/B)) × 100%, where A was the reconstructed volume or height and B was the volume or height of the native orbit. The reconstruction rates and bending of the PDS 0.25 mm and PDS 0.5 mm foils are presented as median values with interquartile ranges. The differences between the reconstruction rates and bending of the PDS 0.25 mm and PDS 0.5 mm foils were analyzed using the Mann–Whitney test.

Statistical significance was assumed when the p-value was < 0.05. The statistical analyses were performed using SPSS version 28 (IBM, New York, USA). The differences between the initial, fractured, and reconstructed orbital volumes and heights when PDS 0.25 mm or PDS 0.50 mm foils were used were visualized using GraphPad Prism version 9 (GraphPad Software, San Diego, USA).

## Results

### Baseline characteristics

Parameters are indicated as median values (with interquartile ranges). The differences between the PDS 0.25 mm and PDS 0.5 mm parameters were analyzed using the Mann-Whitney test.

The orbital defect area, orbital floor area, initial orbital volume and height, fractured orbital volume and height, and reconstructed orbital volume and height did not significantly differ between the orbits that were reconstructed with either PDS 0.25 mm or PDS 0.5 mm. Further details of the baseline characteristics are listed in Table 1.

**Table 1:** Orbital characteristics of 11 fresh frozen cadaver heads with isolated orbital floor fractures

Variable	*Orbits reconstructed with PDS 0.25 mm (n = 11)*	*Orbits reconstructed with PDS 0.5 mm (n = 11)*	*p* value
Orbital defect area (mm^2^)	444.2 (102.2)	462.7 (123.8)	0.8977
Orbital floor area (mm^2^)	859.7 (158.9)	848.9 (118.2)	> 0.9999
Initial orbital volume (mm^3^)	27,567 (5,718)	27,888 (6,721)	0.7477
Fractured orbital volume (mm^3^)	29,156 (5,365)	28,841 (6,536)	0.8470
Reconstructed orbital volume (mm^3^)	27,110 (5,683)	27,059 (5,557)	0.8470
Initial orbital height (mm)	34.73 (2.56)	34.20 (3.30)	0.4099
Fractured orbital height (mm)	38.90 (2.84)	38.40 (2.60)	0.4385
Reconstructed orbital height (mm)	33.17 (3.64)	32.37 (1.83)	0.1206

### Orbital volume in native, fractured, and reconstructed orbits

**Fig. 4:**
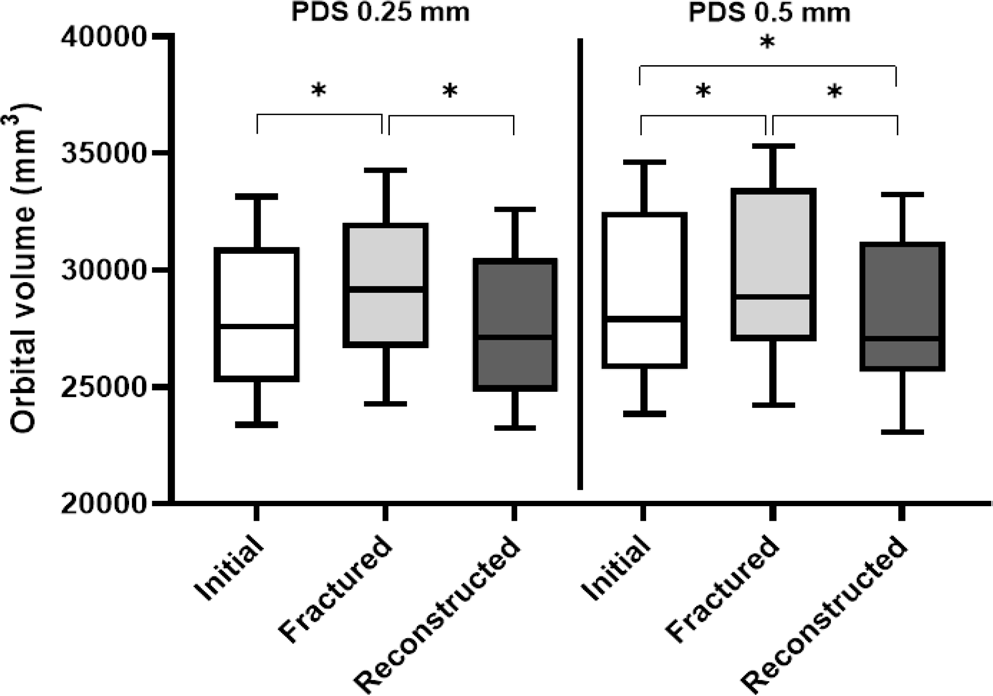
Orbital volume. Box plot showing the orbital volumes (mm^3^) of the initial, fractured, and reconstructed orbits. The data for orbits reconstructed using PDS 0.25 mm and PDS 0.5 mm are shown on the left and right, respectively. The p-values correspond to the differences between the orbital volumes of the initial, fractured, and reconstructed orbits analyzed using the Wilcoxon matched-pairs signed-rank test

In both groups, the orbital volume significantly increased after the creation of the isolated orbital floor fracture (PDS 0.25 mm: *p* = 0.001; PDS 0.5 mm: *p* = 0.001) and significantly decreased after the orbital floor reconstruction (PDS 0.25 mm: *p* = 0.001; PDS 0.5 mm: *p* = 0.001). In the orbits that were reconstructed using PDS 0.5 mm, the post-reconstruction orbital volume was significantly (*p* = 0.0137) smaller than the initial orbital volume; however, there was no significant difference between the post-reconstruction and initial orbital volumes when PDS 0.25 mm was used (Fig. 4).

### Orbital height in native, fractured, and reconstructed orbits

**Fig. 5:**
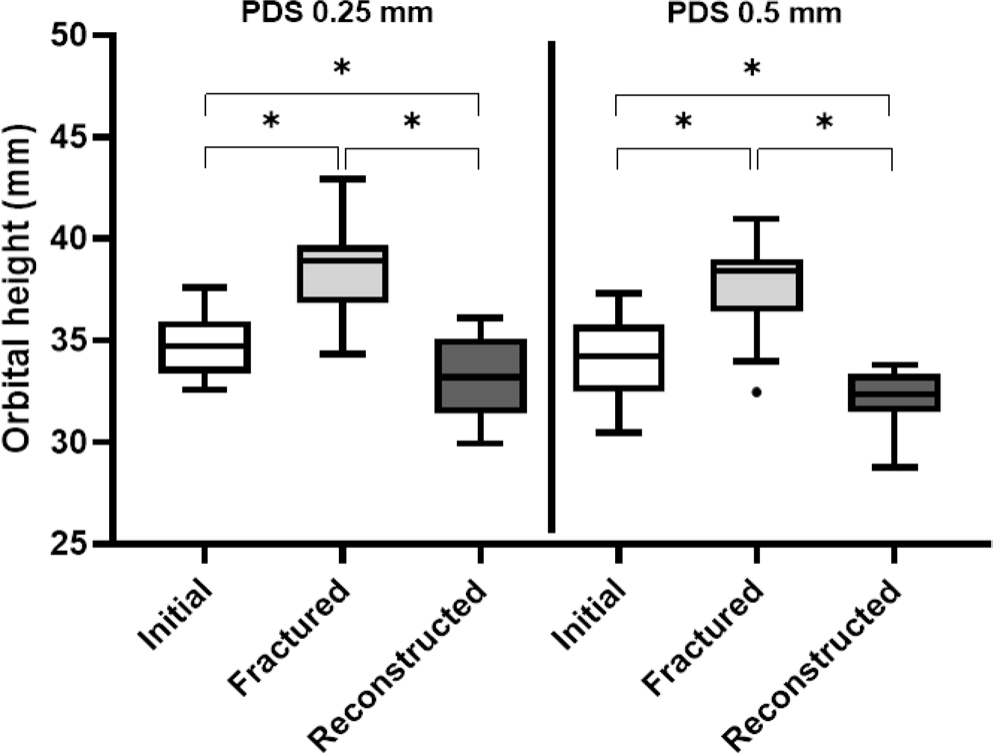
Orbital height. Box plot showing the orbital heights (mm) of the initial, fractured, and reconstructed orbits. The data for orbits reconstructed using PDS 0.25 mm and PDS 0.5 mm are shown on the left and right, respectively. The p-values correspond to the differences between the orbital heights of the initial, fractured, and reconstructed orbits analyzed using the Wilcoxon matched-pairs signed-rank test

In both groups, the orbital height significantly increased after the creation of the isolated orbital floor fracture (PDS 0.25 mm: *p* = 0.001; PDS 0.5 mm: *p* = 0.002) and significantly decreased after the orbital floor reconstruction (PDS 0.25 mm: *p* = 0.001; PDS 0.5 mm: *p* = 0.001). The orbital heights of the orbits reconstructed with PDS 0.25 mm (*p* = 0.001) or PDS 0.5 mm (*p* = 0.001) were significantly less than those of the initial orbits (Fig. 5).

### Discrepancies in the orbital volume and orbital height and foil bending after reconstruction

The median percentual discrepancy from the initial orbital volume was 1.4% when the orbit was reconstructed with PDS 0.25 mm and 2.6% when the orbit was reconstructed with PDS 0.5 mm. Overcorrection of the orbital volume occurred after reconstruction with both PDS 0.25 mm and PDS 0.5 mm, with no significant difference between the methods (*p* = 0.4779).

The median percentual discrepancy from the initial orbital height was 4.0% when the orbit was reconstructed with PDS 0.25 mm and 5.4% when the orbit was reconstructed with PDS 0.5 mm. Overcorrection of the orbital height occurred after reconstruction with both PDS 0.25 mm and PDS 0.5 mm, with no significant difference between the methods (*p* = 0.5619).

While the median bending of the PDS 0.25 mm was 2.3 mm, the median bending of the PDS 0.5 mm was 1.0 mm. No significant difference (*p* = 0.3535) was observed between PDS 0.25 mm and PDS 0.5 mm in terms of bending behavior (Table 2).

**Table 2:** Reconstruction rates of the orbital height and volume and the foil bending of PDS 0.25 mm and PDS 0.5 mm

Variable	*Orbits reconstructed with PDS 0.25 mm (n = 11)*	*Orbits reconstructed with PDS 0.5 mm (n = 11)*	*p* value
Orbital volume correction rate (%)	101.4 (2.0)	102.6 (3.0)	0.4779
Orbital height correction rate (%)	104.0 (6.4)	105.4 (6.7)	0.5619
Foil bending (mm)	2.3 (3.1)	1.0 (1.3)	0.3535

Parameters are indicated as median values (with interquartile ranges). Correction rates were calculated according to Wi et al. [25]. The differences between the PDS 0.25 mm and PDS 0.5 mm parameters were analyzed using the Mann-Whitney test.

## Discussion

This study evaluated the potential of PDS foils with different thicknesses for orbital floor reconstruction through the use of 22 cadaver orbits. The advantages associated with performing orbital reconstruction using resorbable alloplastic materials (such as PDS foils) include the immediate availability of material, no morbidity at a donor site, and the capacity of the material to support the orbital contents via the formation of an autologous soft tissue scar as it degrades [10, 11]. Compared to thicker PDS foil material, the use of thinner PDS foil material can reduce postoperative complications [19, 21], such as the extent of foreign body reactions, and thinner materials have a higher bending capacity due to their lower flexural stiffness [26], which theoretically corresponds more closely to orbital funnel geometry.

Regardless of its capacity to reconstruct the geometric specifications of the native orbit, the biomechanical stability of PDS foil material in the presence of extended orbital floor defects continues to be debated. Assuming that PDS foil material serves as a temporary, non-load-bearing barrier between the orbital and sinus compartments and not as a load-bearing retention [11], some scholars are concerned that there is inadequate support for the orbital contents after the resorption of the foil material, which increases the risk of developing diplopia and late enophthalmos when the defect size exceeds the critical threshold of 250–300 mm^2^ [13, 16–18]. However, the use of PDS foil material in the reconstruction of defects with a median size of 432.0 mm^2^ [5] and isolated orbital floor defects of up to 1019.0 mm^2^ [19] without the development of secondary complications attests to the need for an in-depth discussion of the biomechanical principles that apply when reconstructing defects of various sizes.

The orbital content weight is estimated to be 30 g or 0.3 N [21, 23, 24]. With a median orbital floor defect size of 450.7 mm^2^ in this study, the repositioned orbital contents theoretically acted with a static mechanical force of 0.000666 MPa (N/mm^2^) on the inserted reconstruction material [20, 23]. The initial puncture strength of a 0.15 mm thick PDS foil was experimentally found to be 2.57 MPa, which corresponds to three times the puncture strength of the native orbital floor [20]. Hence, for a defect with a median area of 450.7 mm^2^ (as tested in this study), a 0.15 mm thick PDS foil would theoretically still have a load-bearing capacity of 0.01149 MPa eight weeks after insertion [21, 24]. Given this discrepancy between the actual force of the orbital contents and the puncture strength of the inserted foil material [21, 23, 24], it may be argued that the resorbable foil material in this case was not merely a separation barrier [11], as the potential load-bearing capacity of the PDS foils did not appear to be fully exhausted. These biomechanical findings indicate that the stability of both the 0.25 mm and 0.5 mm foils was sufficient for initially bridging the extended orbital floor defects created in the current study; conventional and pre-bent titanium meshes or patient-specific implants would also have been suitable [27–29].

An inherent limitation of this study, and other cadaveric studies, is that the orbital contents differed from those in real living tissues, demonstrating lower elasticity, inconstant bulbus pressure, postmortem ocular degeneration and hypotrophy, and an inability to generate negative pressure in the paranasal sinuses [21, 23], which cannot be fully countered by manual re-tensioning of the eyeball to 30 g. The influence of dynamic forces and time on the resorbable reconstruction material [20–22] was not considered in this study, as this work focused on comparing the reconstructive accuracy of two different PDS foil thicknesses for the repair of standardized orbital floor defects.

Orbital floor reconstruction must not only meet biomechanical needs but also ensure the accurate restoration of the orbital geometry. Since orbital volume and orbital height determine the bulbus position and ensure facial harmony, these parameters are used to clinically assess the sufficiency of the initial reconstructive geometry [4, 5, 16]. After trauma, both orbital volume and height increase, as the orbital contents herniates through the defect site into the maxillary sinus [25, 30–32]. Treatments for primary orbital floor fractures are generally designed to result in a true-to-original reconstruction of the orbital geometry [33], and the outcomes of such treatments have steadily improved with the introduction and development of patient-specific implants [29]. However, other studies have reported no difference in volumetric reconstructive accuracy at 12 weeks [28, 34] and six months [35] in patients with favorable and unfavorable clinical outcomes. Instead, volumetric tolerance [31] appears to depend primarily on the direction of the trend in volume deviation and the timing of orbital floor reconstruction. An increase in the orbital volume, due to herniation of orbital tissue after trauma [36, 37] or deformation of resorbable foil material after reconstruction of the orbital floor [32], has been reported to carry an increased risk for the development of enophthalmos. Whereas a decrease in the orbital volume (i.e., overcorrection of the orbital geometry) might reflect the need for secondary reconstructive procedures [38], which are required in 0.4% of cases after initial treatment of orbital blowout fractures [39] and are intended to camouflage soft tissue deficits [40] or compensate for a relapse in globe projection after correction of enophthalmos [41]. Overcorrection of the orbital geometry in the clinical context of primary treatment of orbital floor defects is largely underrepresented in the literature but is a known phenomenon in cadaveric studies [42, 43], to which the abovementioned limitations of cadaveric studies apply.

In this cadaveric study, overcorrection of the orbital height and volume were observed, regardless of the PDS foil material thickness. The vertical orbital height after orbital floor reconstruction is influenced by the bending behavior of the PDS foil material, which is initially determined by the force that the bulbus applies to the reconstruction material as a function of the orbital defect area [20, 23]. Compared to the initial bending of a 0.15 mm thick PDS foil [21, 24], the PDS 0.25 mm and PDS 0.5 mm foils were found to bend more despite their higher flexural stiffness [26], and this may be attributed to the larger median orbital defect area of 450.7 mm^2^ used in this study. Although the use of PDS 0.25 mm and PDS 0.5 mm resulted in similar degrees of overcorrection of the orbital height and foil bending, PDS 0.25 mm appeared to bend more and tended to restore orbital height more accurately than PDS 0.5 mm. In terms of the orbital volume, overcorrection that resulted in a significant difference between the reconstructed and native orbital volumes was observed when PDS 0.5 mm was used. However, the use of PDS 0.25 mm resulted in a restored orbital volume that was not significantly different from the initial volume. In summary, based on the restored orbital volume consistency and the quantitatively higher reconstructive accuracy of the orbital height and orbital volume, PDS 0.25 mm appeared to provide superior reconstructive results compared to PDS 0.5 mm in this study. As an inherent limitation of a study on cadaveric specimens, this study is blind to the occurrence of clinical symptoms following orbital reconstruction. Therefore, this study cannot make any statements about post-reconstructive complications, such as persistent double vision or enophthalmos, the occurrence of which would be decisive for the indication to perform secondary reconstructive measures [39]. Instead, this study deliberately investigated the geometric accuracy of orbital reconstruction using resorbable implants with different thicknesses. For this purpose, vertical height and orbital volume were used as surrogate parameters of orbital geometry, as vertical height and orbital volume define facial harmony [4, 5, 16]. Contrary to the surgical intuition to use thicker foil material to safely bridge the bony defect area, this study on cadaver specimens may allow the clinical translation to treat isolated orbital floor fractures with thinner foil material to ensure accurate restoration of facial harmony when there is an indication for the use of resorbable orbital implants.

The findings of this study also indicated that the use of PDS tended to result in an overcorrection of the orbital geometry. This could have been caused by a mismatch between the flat design of the foil and the convex–concave shape of the orbital floor. A discussion of different orbital implants for reconstruction of vertical height and orbital volume must consider the funnel geometry of the orbit and the anatomically complex, convex-concave shape of the orbital floor [44]. With resorbable foil material, the orbital height and the orbital volume seem to adapt naturally as the repositioned orbital contents naturally act on the flat and flexible foil material [23] and bend it depending on the foil thickness, as shown by the results of this study. Perforated or thermoplastic resorbable alloplastic material might be more shapable and therefore correspond more closely to the native orbital geometry [4, 45, 46].

Conventional titanium meshes must be bent by hand and might therefore lead to inaccuracies in the reconstruction of the vertical height and orbital volume [47]. Reconstruction with patient-specific orbital implants is based on the idealistic assumption that the vertical height and the orbital volume are identical on both sides, as the patient-specific implant corresponds to the mirror-image geometry of the orbital floor of the contralateral, healthy side [28, 48]. Ultimately, a disadvantage of rigid orbital implants could result from the fact that rigid orbital implants do not appear to allow natural adaption of the vertical height and orbital volume after reconstruction, but might instead permanently dictate the parameters of the orbital geometry due to the rigidity of the reconstruction material directly from the time of their insertion into the situs.

In terms of procurement, resorbable and standard preformed orbital implants are less expensive than individualized, patient-specific implants [28, 49]. In particular, due to the often comparable outcomes between expensive and cheap orbital implants, cost efficiency must be considered in order to conserve healthcare resources in the future [50]. Future research on resorbable reconstructive materials should focus on the development of thinner PDS foil material for the repair of small isolated orbital floor fractures [21] and posterior-impacted γ-shaped implants to prevent dislocation of resorbable reconstructive material in extended fractures [51].

## Conclusion

In this study, extended, isolated orbital floor fractures reconstructed using PDS foil material demonstrated overcorrection of the orbital geometry regardless of the foil thickness. Due to its lower flexural stiffness, PDS 0.25 mm appeared to provide more accurate reconstruction of the orbital geometry than PDS 0.5 mm, although no significant difference was observed in the reconstructive accuracy between PDS 0.25 mm and PDS 0.5 mm in this cadaveric study.

## Data Availability

Data are available from the corresponding author upon reasonable request.
